# Clinical Study of Three-Dimensional Laparoscopic Partial Nephrectomy for the Treatment of Highly Complex Renal Tumors with RENAL Nephrometry Scores of ≥10 Points

**DOI:** 10.1155/2020/5824209

**Published:** 2020-12-18

**Authors:** Maomao Li, Yu Ren, Guobin Weng

**Affiliations:** Department of Urologic Surgery, Ningbo Urology and Nephrology Hospital, Ningbo, Zhejiang 315100, China

## Abstract

**Aims:**

To examine the safety and feasibility of three-dimensional (3-D) laparoscopic partial nephrectomy for clinically complex renal tumors.

**Materials and Methods:**

We retrospectively evaluated 76 patients who underwent a 3-D (*n* = 42; age, 54.6 ± 12.2 years) or two-dimensional (2-D) laparoscopic partial nephrectomy (*n* = 34; age, 54.8 ± 13.2 years) for renal tumors with RENAL nephrectomy scores of ≥10 points from the same surgical group between January 2017 and April 2020 in Ningbo Urology and Nephrology Hospital. Mean tumor diameter, operation time, warm ischemic time, amount of intraoperative blood loss, postoperative hospitalization time, hospitalization cost, perioperative complication rate, and renal function were compared.

**Results:**

The operation time (154.6 ± 45.1 min) and warm ischemic time (22.5 ± 6.8 min) in the 3-D laparoscopic group were significantly lower than those in the 2-D laparoscopic group (193.0 ± 59.2 min, *p* = 0.001 and 28.7 ± 7.8 min, *p* = 0.0002, respectively). No significant differences in amount of intraoperative blood loss (*p* = 0.642), length of postoperative hospital stay (*p* = 0.541), perioperative complication rate (*p* = 0.860), total hospital cost (*p* = 0.641), and renal function changes including estimated glomerular filtration rate and serum creatinine (*p* > 0.05) were found between the two groups.

**Conclusion:**

Our preliminary experience showed that the 3-D laparoscopic imaging system significantly shortened the operation and renal ischemic times, which are more conducive to partial resection of highly complex renal tumors.

## 1. Introduction

Renal cell carcinoma (RCC) is the most common malignant tumor of the renal parenchyma, accounting for 90% of all cases [1]. It is one of the 10 most common types of cancer in men. Its incidence rate has been increasing continuously in recent years [2]. Nephron-sparing surgery (NSS) has been recommended by urology guidelines as a standard surgical procedure for T1a renal cancer [3]. When conditions are feasible, some T1b stage renal cancers can also be treated with NSS [4]. The RENAL nephrometry score was used to evaluate the spatial anatomical characteristics of renal tumors, which provided a standardised basis for surgeons' decision to perform NSS and for clinical research [5]. In recent years, three-dimensional (3-D) laparoscopic technology has been widely used in surgery. Several studies have shown that 3-D laparoscopic technology has significant advantages of precise removal of kidney tumors, good protection of kidney function, and effective prevention and control of complications [6, 7]. However, reports of NSS cases for highly complex renal tumors are still few. In this study, we retrospectively evaluated 76 patients with RCC who underwent 3-D (*n* = 42) and two-dimensional (2-D) laparoscopy (*n* = 34) between January 2017 and April 2020 who had RENAL nephrometry scores of ≥10 points. We compared operation time, renal artery occlusion warm ischemic time, amount of intraoperative blood loss, length of postoperative hospital stay, hospitalization cost, and perioperative complication rate. The purpose of this study was to examine the efficacy and safety of 3-D laparoscopic partial nephrectomy for highly complex renal tumors.

## 2. Materials and Methods

### 2.1. Clinical Data

**Figure 1:**
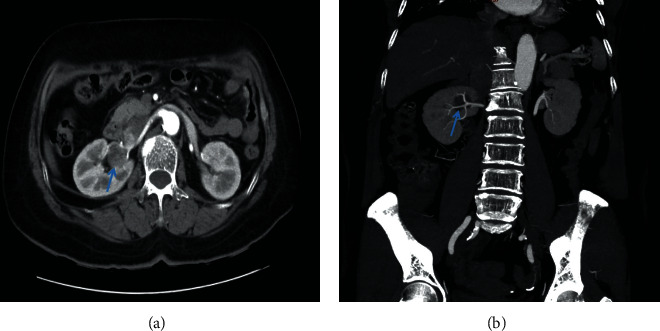
The CT image of a complex renal tumor. (a) CT coronal section. (b) CT transverse section.

A retrospective analysis of the clinical data of 76 patients who underwent laparoscopic partial nephrectomy was performed by the same operation group in Ningbo Urology and Nephrology Hospital between January 2017 and April 2020, including 42 cases (32 males and 10 females, collected from June 2018 to April 2020) in the 3-D laparoscopy group and 34 cases (20 males and 14 females, collected from January 2017 to June 2018) in the 2-D laparoscopy group. The physicians in the operation group had more than 10 years of experience (more than 500 patients) in the laparoscopic operation. All the enrolled patients underwent physical examination and had no other symptoms. Preoperative imaging (chest radiography, ultrasonography, and computed tomography [CT]) examination excluded local infiltration or distant metastasis, and the diagnosis was single renal tumor (of a typical height). The CT image of a complex renal tumor is shown in Figure 1. This study was approved by the medical ethics committee of Ningbo Urology and Nephrology Hospital, and all the patients signed an informed consent form.

### 2.2. Surgical Equipment

The surgical equipment used for the 3-D laparoscopy group was a high-definition 3-D laparoscopy system (Karl Storz, Tuttlingen, Germany) that included a 30° side viewing and 180° reversible 3-D laparoscope. The laparoscope chosen for the 2-D laparoscopy group was a 30° high-definition 2-D laparoscopic surgical system (Karl Storz). All the surgical instruments used were traditional laparoscopic surgical devices.

### 2.3. Transabdominal Partial Nephrectomy

The patient was placed in a 70–90° healthy side-lying position after general anesthesia. After sterilization and towel laying, the rectus abdominis was incised approximately 2 cm above the umbilicus. After establishing a pneumoperitoneum, a 10 mm trocar and observation mirror were inserted. Then, a 12 mm trocar was placed at the midline of the subclavian clavicle and anterior axillary line, approximately 2 cm above the anterior iliac spine. Laparoscopic surgical instruments were placed. The posterior abdominal cavity was accessed through Toldt's gap. Then, the perirenal fascia was opened to free and expose the renal pedicle and renal tumor.

The renal artery was blocked with a bulldog vascular clip, and the renal tumor was removed with an ultrasonic knife at a distance of approximately 0.5–1.0 cm from the tumor. The inner layer was sutured with 3-0 absorbable barb sutures, and then a 2-0 absorbable barb suture was used to close to suture the renal parenchyma continuously. A Hem-o-lok clip was attached at the tip and end of the thread to prevent slippage. After opening the blood supply to the renal artery, bleeding of the suture wound was observed. The tumor was removed, then a drainage was placed, and the incision wound was closed. The patients were bedridden after surgery.

### 2.4. Retroperitoneal Partial Nephrectomy

The patient was placed in a healthy side-lying position after general anesthesia. A 1.5 to 2.0 cm incision was made above the iliac crest of the midaxillary line. The blunt free fingers established the retroperitoneal cavity. Then, a pneumoperitoneum was established by placing a cannula horizontally under the costal margin of the posterior and anterior axillary lines. The renal fascia was cut free, exposing the tumor. Then, the renal artery was freed, and the renal artery was blocked with an arterial clip. The tumor was removed with a 0.5 to 1.0 cm resection margin. The inner layer was sutured with 3-0 absorbable barb sutures, and then a 2-0 absorbable barb suture was used to suture the renal parenchyma continuously. A Hem-o-lok clip was attached at the tip and end of the thread to prevent slippage. After opening the blood supply to the renal artery, bleeding of the suture wound was observed. A drainage was placed, and the incision wound was closed. The patients were bedridden after surgery.

### 2.5. Statistical Analyses

The SPSS 20.0 software (IBM Corp, Armonk, NY, USA) was used for data processing. The measurement data are expressed as mean ± standard deviation. The clinical data of the two groups were compared using a *t* test, and the countable data were tested using the chi-square test. For the data that did not conform to the normal distribution, logarithmic transformation was used to reduce the skewness distribution or the Kruskal Wallis test which was finally used to calculate. A two-tailed *p* value of <0.05 was considered significant.

## 3. Results

### 3.1. Clinical Data

**Figure 2:**
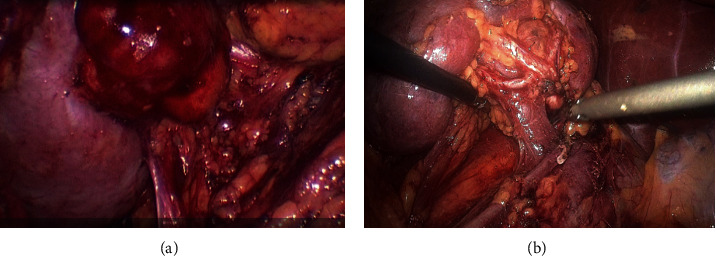
Comparison of the surgical field of 3D laparoscopy and 2D laparoscopy. (a) Field of view in 2D laparoscopic surgery. (b) Field of view in 3D laparoscopic surgery.

The general information of the two patient groups is shown in Table 1. No statistically significant differences in the various parameters were found between the groups (*p* > 0.05). As shown in Figure 2, the 3-D laparoscopy had a clearer view and more precise anatomy than 2-D laparoscopy during surgery. The perioperative clinical indicators of the patients in the 3-D and 2-D laparoscopy groups are shown in Table 2. The operation time (3-D vs. 2-D: 154.6 ± 45.1 min vs. 193.0 ± 59.2 min, *p* = 0.001) and renal artery ischaemic time (3-D vs. 2-D: 22.5 ± 6.8 vs. 28.7 ± 7.8, *p* = 0.0002) in the 3-D laparoscopy group were shorter than those in the 2-D laparoscopy group. We found no significant differences in the amount of intraoperative blood loss (*p* = 0.642), length of postoperative hospital stays (*p* = 0.541), hospital cost (*p* = 0.641), and perioperative complication rate (*p* = 0.860) between the two groups. The changes in the renal function before and after operation were summarized in Table 3. No significant differences were found in the preoperative estimated glomerular filtration rate (eGFR) (*p* = 0.798), postoperative eGFR (*p* = 0.709), eGFR changes (*p* = 0.124), preoperative serum creatinine (*p* = 0.438), serum creatinine statuses at 24 hours (*p* = 0.112), and 3 months after operation (*p* = 0.374). Two patients showed elevated serum creatinine levels, which were maintained in the compensatory period of renal insufficiency (serum creatinine level, <178 *μ*mol/L). The patient was asymptomatic and did not receive special treatment.

### 3.2. Postoperative Pathological Conditions

The postoperative pathological findings of all the patients showed no cases of positive resection margin. As shown in Table 4, the 3-D laparoscopic group had 34 cases of clear cell carcinoma, three cases of chromophobe cell carcinoma, and four cases of renal vascular smooth muscle lipoma. The 2-D laparoscopic group had one case of renal eosinophilia, 27 cases of clear cell carcinoma, three cases of chromophobe cell carcinoma, and four cases of renal angiomyolipoma. The follow-up period ranged from 1 to 36 months. None of the patients had a tumor recurrence, an incision implantation, or a distant metastasis. All the patients survived.

## 4. Discussion

Recent studies have confirmed that partial and radical nephrectomies have similar results on tumor progression-free survival, but NSS can significantly improve patients' quality of life. Therefore, NSS is the first choice of treatment for T1 stage renal tumors [3]. The 3-D laparoscope can provide high-definition 3-D stereo vision and a surgical field of vision closer to reality and more clearly define the boundary between the tumor and normal tissues. Surgeons can more accurately remove the tumor, maximise the preservation of the renal parenchyma of the affected kidney, and reduce the initial technical difficulty of learning the surgical technique to shorten the learning curve of residents. Therefore, 3-D laparoscopy is more conducive to NSS as a minimally invasive treatment for complex renal tumors [8].

In the treatment of highly complex renal tumors, the application of NSS is restricted by the anatomical characteristics and complexity of the tumor. Renal tumors with RENAL scores of ≥10 points are generally large and deep lying, which make the boundaries of the tumors difficult to determine during surgery. In this study, patients with renal tumors with RENAL scores of ≥10 points were selected as research subjects, and partial laparoscopic nephrectomy was performed. The purpose was to use 3-D laparoscopy to reduce the intraoperative warm ischemic time, accurately and completely remove the tumor, and ensure good postoperative kidney function. Many studies have reported that warm ischemic times are closely related to the development of the renal function impairment after NSS. Therefore, knowledge on how to improve the wound suture technique and shorten the warm ischemic time is key to the success of NSS [9]. A previous study showed that a warm ischemic time of >25 minutes after renal artery occlusion will cause aggravation of renal function damage, and for every 1 minute increase in warm ischemic time, the patient's risk of acute renal failure after surgery increases by 5%, which leads to stage IV chronic kidney disease. This increases the risk of renal insufficiency by 6% [10, 11]. Therefore, reducing the warm ischemic time is important in laparoscopic partial nephrectomy. Kunert et al. [12] also found that the 3-D laparoscope can quickly and accurately adjust the angle of the needle into and out of the target tissue when suturing the renal parenchyma. It allows for quick completion of the suture and tying of the knot, reduces the blocking time of the renal artery, and effectively protects kidney function. 3-D laparoscopy can overcome the limitation of the operator's lack of stereoscopic perception of the depth of the operation during traditional laparoscopy, which reduces the difficulty of complicated operations, such as sutures and knots during operation, and shortens the warm ischemic time accordingly. In this study, the warm ischemic times ranged from 16 to 38 minutes, with a mean of (22.5 ± 6.8) minutes, which was within the controllable range.

However, clinical studies have shown a clear correlation between kidney volume and renal function, and the amount of retained renal parenchymal volume is also an important influencing factor of postoperative renal function recovery [13]. Lane et al. [14] reported that in patients with kidney cancer, the older they are or the more basic their diseases before surgery, the worse their renal reserve function and the greater the kidney function decline after surgery. Woldu et al. [15] also confirmed that the renal parenchyma of the remaining kidney after operation can compensate for the function of the nephrectomy unit. Our study revealed that the creatinine level was significantly higher at 24 hours after operation than before operation and decreased significantly at 3 months after operation. This situation may be the compensatory mechanism of the remaining kidney of the postoperative patient and the start of the reserve function of the kidney itself, which can compensate for the lost kidney function. CT urography was performed at 3 months after the operation for both kidneys, indicating that the affected kidneys were all functional. Reexamination of the renal function at 3 months revealed no significant difference in the blood creatinine level. This also shows that this kind of reserve function of the kidney itself makes it relatively stable in the short-term after renal function recovery.

With the widespread promotion of NSS, its value and advantages in treating early renal tumors are increasingly accepted by urologists and patients. The size and growth location of the tumor are closely related to the success rate of NSS. Therefore, the RENAL scoring system has a good guiding role in evaluating the complexity of the operation. The results of this study show that 3-D laparoscopy has greater advantages than 2-D laparoscopy: ① it can increase the speed of surgery and shorten the operation time. ② it can shorten the renal artery occlusion time. In this study, the perioperative complication rate was 7.1% (3/42) in the 3-D laparoscopic group and 8.8% (3/34) in the 2-D laparoscopic group. All the patients improved after conservative symptomatic treatment, and no serious perioperative complications occurred. The results were similar to the perioperative complications reported in previous studies of partial nephrectomy [16, 17]. With 3-D laparoscopy, surgeons can more accurately and quickly perform precise operations such as grasping and cutting, which leads to shorter operation and renal artery occlusion warm ischemic time than those in the 2-D laparoscopy group. These results also support the previous research [18, 19].

There were some limitations in the present study. Firstly, this study was a single-center, retrospective case analysis, so there was a certain selection bias in the inclusion of cases. Secondly, the surgeon had huge 2D experience which allows them to improve their basic laparoscopic skills such as hand-eye coordination and intraoperative interaction with assistant surgeon using laparosopic camera. Thus, better results should not be explained only by 3D technology. Thirdly, the sample size of our study was small, and future studies needed to further expand the sample size to confirm the results of this study. Fourthly, the hospitalization days in Western countries were only 1-2 days, while the hospitalization days in our study were 7-9 days. This might be due to the Chinese consciousness and Western medical insurance policies. In addition, rapid rehabilitation should be implemented in the course of patient rehabilitation in the future study.

## 5. Conclusions

This study suggests that NSS is safe and feasible for the treatment of highly complex renal tumors with RENAL scores of ≥10 points. The 3-D laparoscopic imaging system significantly shortened the operation and renal ischemic times, which are more conducive to partial resection of highly complex renal tumor.

## Figures and Tables

**Table 1:** Comparison of clinical data between patients with 3D laparoscopy and 2D laparoscopy.

Clinical data	3D group	2D group	*p*
Age(year)	54.6 ± 12.2	54.8 ± 13.2	0.945
Male/female	32/10	20/14	0.170
BMI	24.9 ± 1.3	24.7 ± 1.2	0.492
Left/right	23/19	19/15	0.893
Diameter of tumor^∗^	5.5 ± 1.2	5.3 ± 1.1	0.381
R.E.N.A.L. score for tumors^∗^	10.5 ± 0.5	10.4 ± 0.5	0.549
Radius score^∗^	1.7 ± 0.3	1.6 ± 0.4	0.217
Exophytic/endophytic score	3.0 ± 0.2	3.0 ± 0.1	0.983
Nearness to sinus fat or collecting system score	2.9 ± 0.2	2.9 ± 0.1	0.994
Location in relation to polar lines score	2.9 ± 0.1	2.9 ± 0.2	0.994

^∗^The *p* value was calculated by *t* test after logarithmic transformation. BMI: body mass index.

**Table 2:** Comparison of perioperative results between 3D laparoscopy and 2D laparoscopy in patients with nephron-sparing surgery.

Clinical data	3D group *N* = 42	2D group *N* = 34	*p*
Operation time (min)^∗^	154.6 ± 45.1	193.0 ± 59.2	0.001
Warm ischemic time (min)^∗^	22.5 ± 6.8	28.7 ± 7.8	0.0002
Intraoperative blood loss (ml)^∗^	55.9 ± 22.0	57.8 ± 24.0	0.642
Postoperative hospital stays (d)^∗^	7.9 ± 1.2	8.1 ± 1.5	0.541
Perioperative complication rate	3/42	3/34	0.860
Infection	2	1	
Bleeding	1	2	
Urinary fistula	0	0	
Total hospitalization cost (yuan)	25319.3 ± 2483.1	25619.2 ± 3103.4	0.641

^∗^The *p* value was calculated by *t* test after logarithmic transformation.

**Table 3:** Changes of the renal function before and after operation in the 3D laparoscopy group and 2D laparoscopy group.

Clinical data	3D group	2D group	*p*
Preoperative eGFR (ml/min/1.73m^2^)^∗^	83.4 ± 15.6	84.5 ± 21.6	0.798
Postoperative eGFR (ml/min/1.73m^2^)^∗^	74.9 ± 19.4	75.5 ± 20.1	0.709
eGFR changes (ml/min/1.73m^2^)^#^	8.5 ± 1.2	9.0 ± 1.6	0.124
Preoperative serum creatinine (mg/dl)^#^	72.5 ± 23.6	70.1 ± 23.2	0.438
Serum creatinine at 24 hours after operation (mg/dl)^∗^	86.1 ± 19.5	94.1 ± 23.9	0.112
3 months after operation, serum creatinine (mg/dl)^∗^	74.3 ± 18.2	79.4 ± 31.0	0.374
Number of cases with undeveloped renal CTU 3 months after surgery	0	0	Na

eGFR: estimated glomerular filtration rate. #The *p* value was calculated by Kruskal Wallis test. ^∗^The *p* value was calculated by *t* test after logarithmic transformation.

**Table 4:** Postoperative pathological conditions in 3D laparoscopy group and 2D laparoscopy group.

Postoperative pathological conditions	3D group	2D group	*p*
Clear cell carcinoma	34	27	0.867
Chromophobe cell carcinoma	3	3	0.787
Renal vascular smooth muscle lipoma	4	4	0.752
Renal eosinophilia	1	0	0.579

## Data Availability

The clinical data used to support the findings of this study are available from the corresponding author upon request.
